# Randomness determines practical security of BB84 quantum key distribution

**DOI:** 10.1038/srep16200

**Published:** 2015-11-10

**Authors:** Hong-Wei Li, Zhen-Qiang Yin, Shuang Wang, Yong-Jun Qian, Wei Chen, Guang-Can Guo, Zheng-Fu Han

**Affiliations:** 1 Key Laboratory of Quantum Information, University of Science and Technology of China, Hefei, 230026, China; 2 Zhengzhou Information Science and Technology Institute, Zhengzhou, 450004, China; 3 Synergetic Innovation Center of Quantum Information & Quantum Physics, University of Science and Technology of China, Hefei 230026, China

## Abstract

Unconditional security of the BB84 quantum key distribution protocol has been proved by exploiting the fundamental laws of quantum mechanics, but the practical quantum key distribution system maybe hacked by considering the imperfect state preparation and measurement respectively. Until now, different attacking schemes have been proposed by utilizing imperfect devices, but the general security analysis model against all of the practical attacking schemes has not been proposed. Here, we demonstrate that the general practical attacking schemes can be divided into the Trojan horse attack, strong randomness attack and weak randomness attack respectively. We prove security of BB84 protocol under randomness attacking models, and these results can be applied to guarantee the security of the practical quantum key distribution system.

Quantum key distribution (QKD)^1^ is the art of sharing secret keys between two remote parties Alice and Bob, unconditional security of which is based on the fundamental laws of quantum mechanics. The detailed security analysis has been proved by applying the entanglement distillation and purification (EDP) technology^2^,^3^ and the von Neumann entropy theory^4^,^5^,^6^ respectively. However, unconditional security of the QKD protocol has an important assumption, which requires Alice and Bob have random input numbers to control the classical bit encoding and measurement bases selection, and it can be easily proved that the measurement outcomes will become unsafe if the input random numbers are controlled or known by the eavesdropper Eve. A pair of important elements in practical QKD system is the random preparation and measurement of quantum states. If these procedures are imperfect, which can be perceived as a kind of incomplete randomness, the deviation may be used to perform quantum attacking^7^. More generally, practical attacking schemes can be divided into three different types from the view point of system randomness.

The first type is the Trojan horse attack^8^, where the signal state combining with the Trojan horse state can be assumed to be high dimensional state modulation. Thus, Eve can measure one dimension of the modulated high dimensional state to get all of the secret key information without being discovered.

The second type is the strong randomness attack, where part of the input random numbers are totally controlled or known by the eavesdropper Eve. For example, the multi photon pulses generated by the practical weak coherent light source can be utilized by Eve to perform photon number splitting (PNS) attack^9^,^10^, if the multi photon encoding quantum states are assumed to be known by Eve. Another example is the detector blinding attack^11^,^12^, where Eve can easily mount the man-in-the-middle (MITM) attack by converting the avalanche photodiodes (APDs) into linear mode. The single photon detector has the count iff Bob’s bases selection is equal to Eve, thus the bases selection in Bob’s side are controlled by Eve. More recently, we proposed the probabilistic blinding attack model^13^, where Eve partly applies the blinding attack to avoid being catched by detecting the current parameter, thus part of the bases selection can be assumed to be controlled by Eve correspondingly. In the strong randomness attack model, the final secret key should shrink Eve’s information from the multi-photon pulses to an arbitrary small value. To avoid the PNS attack, Gottesman-Lo-Lutkenhaus-Preskill (GLLP)^14^ To avoid the PNS attack, Gottesman-Lo-Lutkenhaus-Preskill (GLLP)^14^ formula combining with the decoy state method^15^,^16^,^17^ is used in practical QKD systems to eliminate all of the multi photon pulse counting result, and ensure that only the single photon counting events can generate the final secret key. To avoid the probabilistic blinding attack, all blinding counting results should be eliminated, and only the non-blinding counting events can generate the final secret key. In the strong randomness attack model, the secret key rate^18^ formula should be modified to


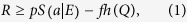


where *p* is the probability of getting valid counting result, which can’t be controlled by Eve. *a* is Alice’s measurement outcome, *E* is Eve’s auxiliary quantum system, *S*(*a*|*E*) = *S*(*a*, *E*) − *S*(*E*) is the conditional von Neumann entropy, *Q* is the practical quantum bit error rate, *h*(*Q*) = −*Q*log_2_*Q* − (1 − *Q*)log_2_(1 − *Q*) is the classical Shannon entropy function, *f* ≥ 1 is the error correction efficiency. If we prove security of BB84 QKD protocol under the PNS attack, *p* and *S*(*a*|*E*) should be estimated by the single photon counting rate and the single photon error rate respectively^14^,^15^,^16^,^17^.

The third type is the weak randomness attack, where the input random numbers are partly controlled by Eve^19^. Such as the wavelength dependence of the beam splitter will introduce the wavelength attack^20^, where Eve can apply different wavelengths to control Bob’s bases selection. Since the practical beam splitter maybe has partial wavelength correlation, that is the coupling ratio can’t reach 0 and 1 with two different wavelengths, thus Eve can only partly control Bob’s bases selection. Another example is the time shift attack^21^, where Eve controls the APDs detection efficiency by controlling the photon arriving time, thus Eve has the advantage to guess the measurement outcomes. Since the practical time shift attack will introduce nonzero error rate, the classical bit encoding can be assumed to be partly known by Eve correspondingly.

Now, the Trojan horse attack can be avoided by applying the dimension filter (such as the wavelength filter) before the state modulation and measurement, which can be utilized to prevent Eve’s Trojan horse light. The strong randomness attacking model has also been analyzed by applying the strict post processing technology, where we only need to precisely estimate *p* and *S*(*a*|*E*). However, the weak randomness attacking model has not been analyzed until now. In this work, we prove security of the practical QKD system with weak input random numbers, which can affect the classical bit encoding and bases selection respectively. We give two security analysis models, the first model is based on the one-step post processing, where all of the measurement outcomes should integrally apply error correction and privacy amplification. While the second model is based on the two-step post processing, where the measurement outcomes can be divided into two sets with different measurement bases, then the two sets should apply error correction and privacy amplification individually. If we only consider the bit encoding weak randomness, two distinct methods can get the same secret key rate. But, if we consider the bases selection weak randomness, the analysis result shows that the two post processing method can generate much more secret key. Our analysis model can be applied in numerous attacking schemes, such as the wavelength attack and the time shift attack. Combining with the previous three attacking models, security of the practical QKD system can be evaluated.

## BB84 QKD Protocol with Weak Randomness

**Figure 1:**
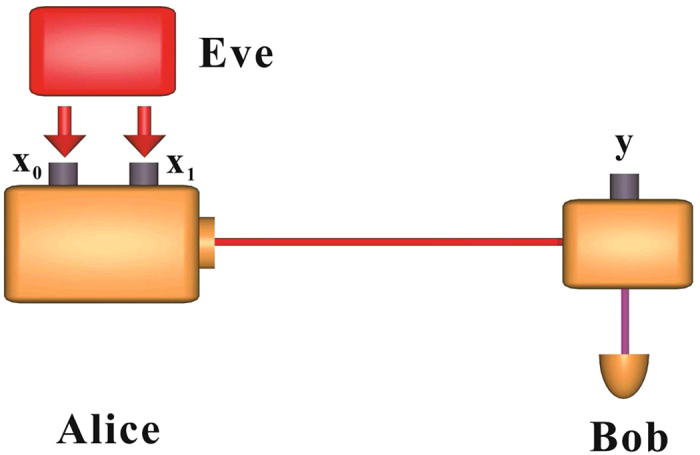
Weak randomness QKD model, where *x*_0_ decides the encoding classical bit, *x*_1_ decides the encoding bases selection, *y* decides the measurement bases selection. In the weak randomness QKD model, Eve has the advantage to guess the classical bit encoding *x*_0_ and the basis selection *x*_1_.

In the BB84 protocol, there are two binary input bits *x*_1_ and *x*_0_ in Alice’s side, which can be used to select the state preparation bases and encoding classical bits respectively. While the state measurement side Bob needs one binary input bit *y* to select the measurement basis. After the quantum state preparation and measurement, Alice and Bob should apply the bases sifting process to save the same bases case (*x*_1_ = *y*). Thus, in the security analysis model, the input randomness can be divided into two sets, the first set can be used to decide the encoding classical bit selection *x*_0_, while the second set can be used to decide the encoding and decoding bases selection *x*_1_ (or *y*). Since Alice and Bob should publicly compare *x*_1_ and *y* to save the same value, we can only consider Eve has partial knowledge about the bases selection *x*_1_ before the state measurement, the security analysis model can be simplified correspondingly. Thus we can only assume weak random numbers *x*_0_ and *x*_1_ to control the encoding classical bit and bases selection respectively, the detailed analysis model is given in Fig. 1.

In the weak randomness model, the weak random numbers *x*_0_ and *x*_1_ can be controlled by two different sets of hidden variables *λ*_0_ and *λ*_1_ as the following equations,


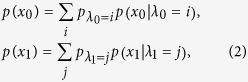


where *λ*_0_ and *λ*_1_ are hidden variables controlled by Eve, *p*(*x*_0_ = 0) is the probability that Alice encodes classical bit 0, while *p*(*x*_0_ = 1) = 1 − *p*(*x*_0_ = 0) is the probability that Alice encodes classical bit 1. Similarly, *p*(*x*_1_ = 0) is the probability that Alice applies the rectilinear encoding basis, *p*(*x*_1_ = 1) = 1 − *p*(*x*_1_ = 0) is the probability that Alice applies the diagonal encoding basis. Note that two sets of hidden variables *λ*_0_ and *λ*_1_ should satisfy 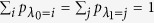. However, even if the practical experimental realization can observe 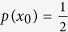 and 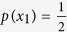 respectively, we still can’t guarantee 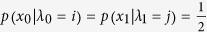 for arbitrary hidden variables *λ*_0_ = *i* and *λ*_1_ = *j*. Thus, the aforementioned security analysis model based on perfect random input numbers can’t be satisfied directly, we need to estimate the randomness deviation for arbitrary hidden variables. The practical weak randomness model is given by


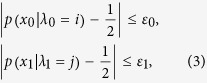


where 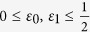, *ε*_0_ = 0 (*ε*_1_ = 0) is the perfect random number case, which means that Eve has no prior knowledge about the classical bit selection (bases selection). While 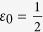 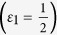 means Eve previously knows the classical bit selection (bases selection), in which case Alice and Bob can’t generate any secret key even if they can observe 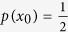 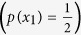.

## One-Step Post Processing Method

By considering the given hidden variable *λ*_0_ = *i*, we apply the EDP technology to illustrate the practical state preparation as the following equation,


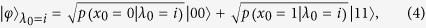


where Alice encoding the classical bit 0 with probability *p*(*x*_0_ = 0|*λ*_0_ = *i*), and encoding the classical bit 1 with probability *p*(*x*_0_ = 1|*λ*_0_ = *i*) = 1 − *p*(*x*_0_ = 0|*λ*_0_ = *i*). By considering the given hidden variable *λ*_1_ = *j*, Alice prepares the quantum state in the rectilinear basis with probability *p*(*x*_1_ = 0|*λ*_1_ = *j*), and prepares the quantum state in the diagonal basis with probability *p*(*x*_1_ = 1|*λ*_1_ = *j*) = 1 − *p*(*x*_1_ = 0|*λ*_1_ = *j*), thus the final quantum state preparation under the Pauli quantum channel is


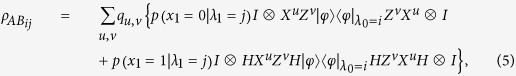


where *u*, *v* ∈ {0, 1}, 
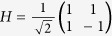
 is the Hadmard matrix, 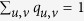, *q*_0,0_ is the probability that Eve applies identity operation 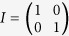, *q*_0,1_ is the probability that Eve applies phase error operation 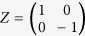, *q*_1,0_ is the probability that Eve applies bit error operation 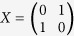, *q*_1,1_ is the probability that Eve applies bit phase error operation *XZ*. Since Alice’s state preparation is restricted in the two dimensional Hilbert space, we can prove the final secret key rate under the Pauli quantum channel. Thus, the quantum bit error rate and phase error rate introduced by Eve can be respectively given by


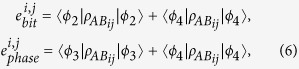


where


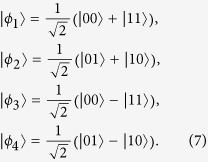


For arbitrary hidden variable *λ*_0_ = *i* and *λ*_1_ = *j*, upper bound of the phase error rate 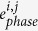 can be estimated by applying the bit error rate 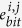 and the randomness deviation parameters,


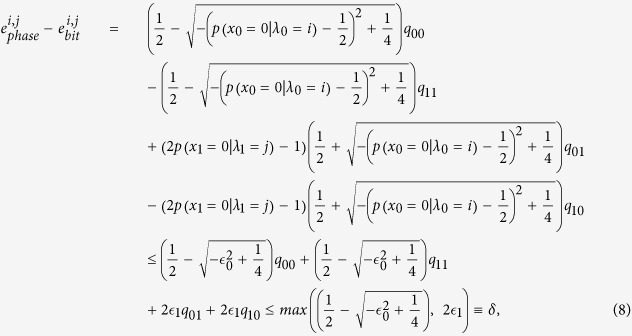


where we apply *q*_00_ + *q*_11_ ≤ 1, *q*_01_ + *q*_10_ ≤ 1 and 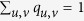 in the previous calculation. By applying the EDP technology, the final secret key rate with given hidden variables *λ*_0_ = *i* and *λ*_1_ = *j* is


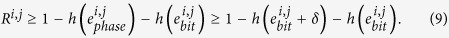


**Figure 2:**
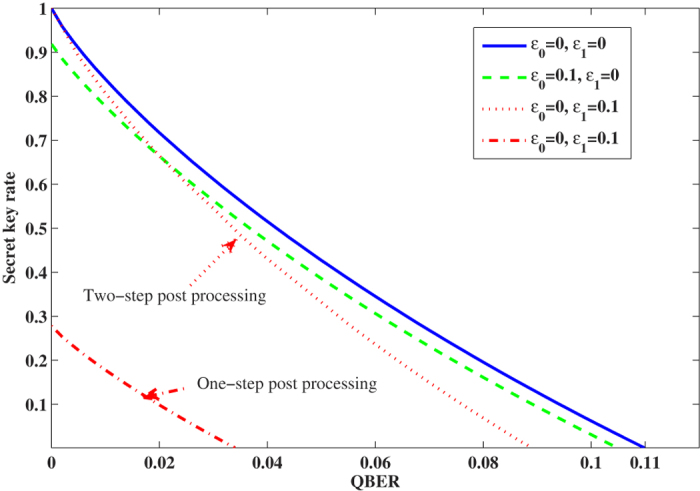
Secret key rate with different quantum bit error rate value, where the blue solid line is no randomness deviation case, the green dash line is considering 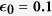 and 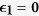, the red dotted line is considering 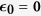 and 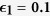 with two-step post processing method, the red dash dotted line is considering 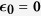 and 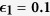 with one-step post processing method. Comparing with the one-step post processing method, two-step post processing method can generate much more secret key with given basis selection randomness deviation, this is because we can get more precious phase error estimation in the two-step post processing method.

In the practical experimental realization, we can only observe the practical quantum bit error rate 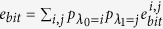, the final secret key rate with given quantum bit error rate *e*_*bit*_ can be given by


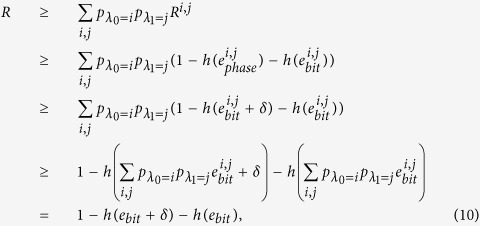


where we apply the concavity property of the Shannon entropy function in the previous calculation. By implementing the security analysis result, we calculate the secret key rate *R* with given randomness deviation parameters 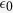 and 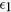 in Fig. 2. The calculation result demonstrates that the bases selection weak randomness decrease the final secret key rate more obviously comparing with the classical bit encoding weak randomness.

## Two-Step Post Processing Method

In the previous weak randomness model, the input random numbers maybe controlled by the hidden variables *λ*_0_ and *λ*_1_. Since there are two different bases selection (diagonal basis and rectilinear basis) and two different classical bit encoding (0 and 1), we can simply assume *λ*_0_ and *λ*_1_ have two different values {0, 1} respectively.

**Figure 3:**
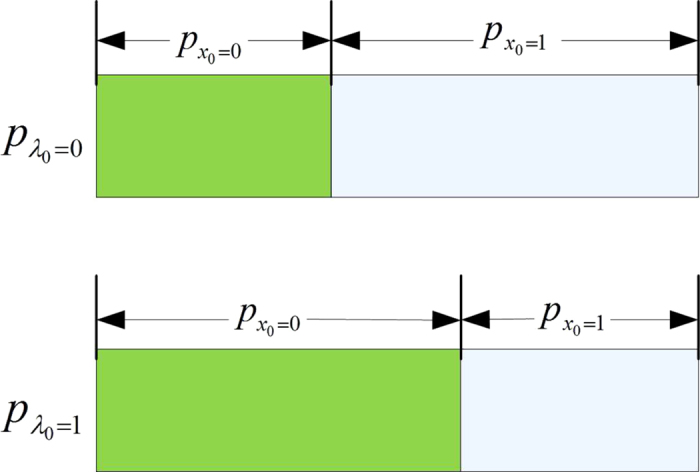
The classical bit encoding *x*_0_ is controlled by the hidden variable *λ*_0_, different *λ*_0_ values have different classical bit encoding probability *p*(*x*_0_|*λ*_0_).

In the practical experimental realization, we can only observe the classical bit encoding probability 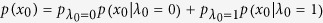, but 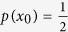 can’t guarantee 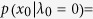 
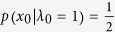
, the detailed classical bit deviation model is given in Fig. 3.

**Figure 4:**
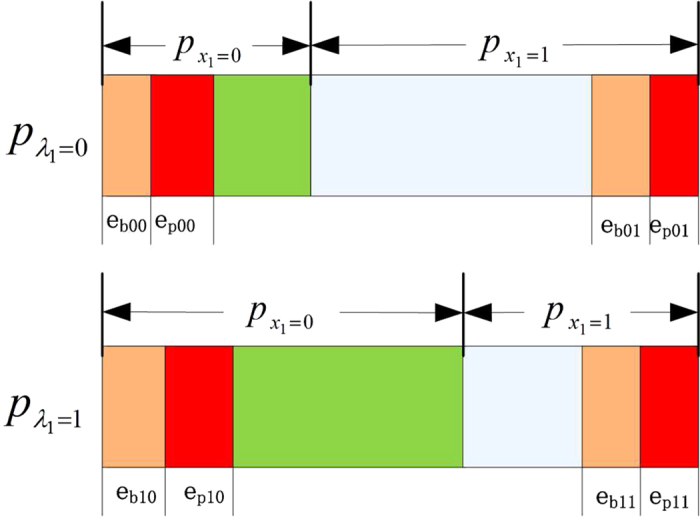
The basis selection deviation is controlled by the hidden variable *λ*_1_, different *λ*_1_ value has different basis selection probability *p*(*x*_1_|*λ*_1_). For given hidden variable *λ*_1_ = 0, *e*_*b*00_ and *e*_*b*01_ are bit error rates introduced in the rectilinear basis and diagonal basis, while *e*_*p*00_ and *e*_*p*01_ are phase error rates introduced in the rectilinear basis and diagonal basis respectively. For given hidden variable *λ*_1_ = 1, *e*_*b*10_ and *e*_*b*11_ are bit error rates introduced in the rectilinear basis and diagonal basis, while *e*_*p*10_ and *e*_*p*11_ are phase error rates introduced in the rectilinear basis and diagonal basis respectively.

Similarly, we can also only observe the bases selection probability 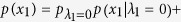 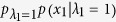, but the observed probability 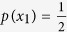 can’t guarantee 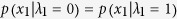 = 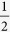, the detailed bases selection deviation model is given in Fig. 4.

The practical quantum state preparation is given by


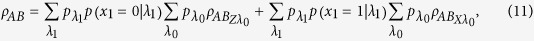


where


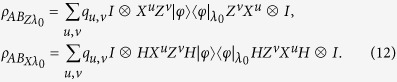


For given hidden variables *λ*_0_ and *λ*_1_, the difference between the phase error rate in the rectilinear basis and bit error rate in the diagonal basis can be given by


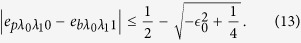


where 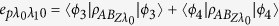, 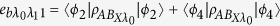. Similarly, The difference between the phase error rate in the diagonal basis and bit error rate in the rectilinear basis can be given by


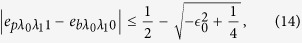


where 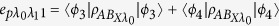, 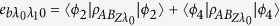. By considering 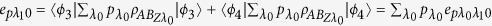 and 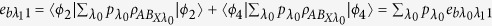, we calculate the difference between the phase error rate 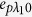 and the bit error rate 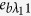



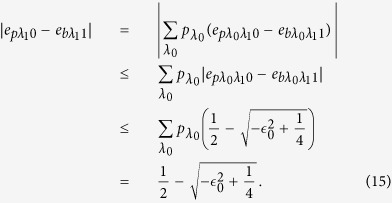


Similarly, the difference between 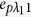 and 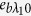 is


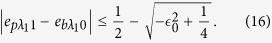


The probability of getting the rectilinear basis and diagonal basis measurement outcomes in Bob’s side can be respectively given by


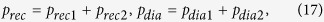


where 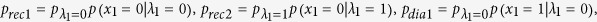 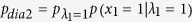. The phase error rate in the rectilinear basis and diagonal basis can be respectively given by


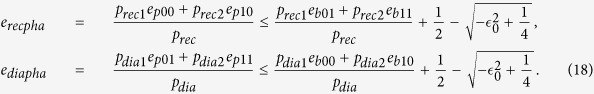


The bit error rate in the rectilinear basis and diagonal basis can be respectively given by


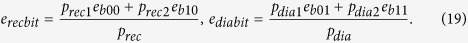


By applying the two-step post processing method with the two different bases measurement outcomes, the final secret key rate can be given by


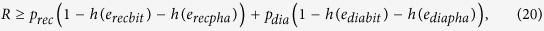


where the first part is the secret key generated by the rectilinear basis, while the second part is the secret key generated by the diagonal basis. The corresponding secret key rate *R* with different quantum bit error rate values is given in Fig. 2, the calculation is based on the nonlinear optimization method with given quantum bit error rate, the detailed explanation is in the methods. To explain our analysis result, we compare the two analysis methods by considering the wavelength attack has the coupling ratio 0.4 and 0.6 with different wavelengths. If the observed quantum bit error rate is 0.02, one-step post processing method can generate the secret key rate 0.0984, while the two-step post processing method can generate the secret key rate 0.6642.

## Methods

By considering Eve’s arbitrary attacking scheme, the final secret key rate with two different bases can be calculated with the following optimization method


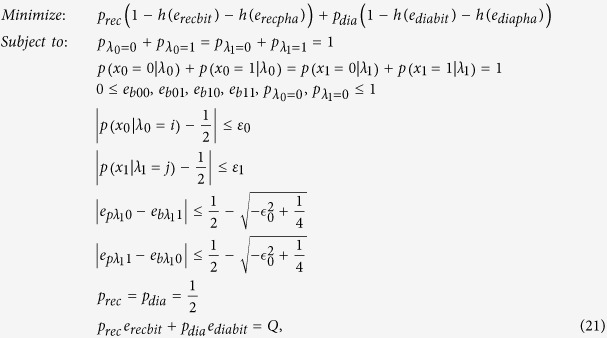


where *Q* is the quantum bit error rate estimated in the practical experimental realization, 
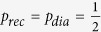
 are the bases selection probability observed in the practical experimental realization.

## Conclusion

In this work, security of BB84 QKD protocol against the strong randomness attack and the weak randomness attack have been analyzed, which satisfies several practical attacking schemes, such as the photon number splitting attack, detector blinding attack, wavelength attack and time shift attack. We demonstrate that security of the practical QKD system can be evaluated by respectively considering the Trojan horse attack, the strong randomness attack and the weak randomness attack, and the three attacking models can be employed to build the practical QKD system security standardization in the future.

## Additional Information

**How to cite this article**: Li, H.-W. *et al.* Randomness determines practical security of BB84 quantum key distribution. *Sci. Rep.* **5**, 16200; doi: 10.1038/srep16200 (2015).
